# Interactions of Oat β-Glucan, Protein, and Starch Under High Hydrostatic Pressure and Their Influence on Starch Properties

**DOI:** 10.3390/gels12050438

**Published:** 2026-05-16

**Authors:** Yangyang Chen, Ying Miao, Rui Huo, Minjun Sun, Jingyu Xie, Meili Zhang

**Affiliations:** College of Food Science and Engineering, Inner Mongolia Agricultural University, Hohhot 010018, China; 15290963005@163.com (Y.C.); miaoying0713@163.com (Y.M.); huorui1@sina.com (R.H.); sun_minjun@163.com (M.S.); xiejingyu2023@163.com (J.X.)

**Keywords:** high hydrostatic pressure, oat starch-β-glucan-protein composite system, physicochemical properties, interaction

## Abstract

Oat starch, β-glucan, and protein are the primary components in oats with high nutritional value, and the interactions among these three constituents markedly influence the starch properties. High hydrostatic pressure (HHP), recognized as a non-thermal processing technique, is primarily employed for the modification of starch and protein in food processing applications. This study aimed to elucidate the interactions among oat β-glucan, protein, and oat starch under 300 MPa HHP treatment and their effects on starch properties. The results showed that at ambient pressure, β-glucan and protein mainly restricted starch swelling and gelatinization through water competition, leading to reductions in pasting viscosity, gelatinization enthalpy, and relative crystallinity. In contrast, HHP treatment significantly enhanced the intermolecular interactions among the three components, thereby improving the freeze–thaw stability, gel elasticity, short-range ordered structure, and thermal stability of the composite system. The study demonstrates that HHP modifies the physicochemical properties of starch by intensifying interactions among its components, providing a theoretical basis and strategy for the development of novel functional starch-based foods using HHP technology.

## 1. Introduction

Naked oats (*Avena nuda* L.) belong to the genus Avena of Gramineae, and their grains are rich in dietary fiber, protein, and unsaturated fatty acids and other bioactive components [1]. Starch, as the main component of oats, accounts for about 60% of the dry weight of the kernel. Oat starch (OS) is significantly different from other cereal starches due to its unique properties, such as high lipid content, small particle size, short amylose chain, and high crystallinity [2]. These properties enable its use as a thickener, stabilizer, and fat substitute in the food field [3]. However, natural starch exhibits limitations such as low thermal stability, easy retrogradation, and poor shear resistance, which restrict its direct application in food processing. For example, Lima et al. [4] reported that OS shows promise for use as an encapsulating; however, structural modification contributes to enhancing the technical performance of this application. Therefore, to meet the diversified needs of food processing, starch modification can be used to enhance its functional properties and explore its new application directions. Starch modification methods mainly include physical, chemical, and enzymatic methods. In recent years, emerging strategies such as complex-based modification, transgenic modification, and nano-modification have further provided green and efficient modification methods. For example, enzymatic extrusion effectively improved the amylose content and thermal stability of oat flour [5]; Chemical modification methods such as octenyl succinic anhydride and lactic acid could effectively increase the pasting viscosity and swelling power of acorn starch, alter its freeze–thaw stability, and improve its thickening performance [6]; The addition of high-amylose corn starch increased the hardness of the wheat starch composite gels [7].

Oat β-glucan (OBG) and protein (OP) are important components of oats. OBG accounts for approximately 3–9% and exhibits processing characteristics such as enhanced viscosity, gelation, and stability, as well as physiological functions including blood glucose reduction and regulation of the gut microbiota [8]. OP accounts for about 11–16% [9], contains eight essential amino acids, and is rich in lysine and threonine, demonstrating high nutritional value and bioactivity. Studies have shown that the hydrolysate of oat bran protein alleviates oxidative stress and neuroinflammation, modulates the gut microbiota in mice, and demonstrates potential as a functional food for delaying aging [10]. Similarly, rice bran peptides have also been found to regulate the gut microbiota and effectively delay the aging process in mice [11]. Furthermore, Non-starch polysaccharides (NSPs) have been demonstrated to regulate starch gelatinization and retrogradation processes, whereas interactions between starch and proteins predominantly govern starch functional properties through hydrogen bonding and electrostatic complexation [12]. For example, OBG suppressed both short- and long-term retrogradation in rice starch [13].

Thermal processing serves as a foundational food technology that facilitates component interactions with operational simplicity. However, elevated temperatures may disrupt starch and protein structures, inducing the Maillard reaction and compromising inherent flavor profiles. Non-thermal modification treatments are combined with other methods to minimize the adverse effects of heat treatment. High hydrostatic pressure (HHP), classified as a non-thermal processing technique, fundamentally differs from conventional thermal methods. Hydrostatic pressure application disrupts or alters non-covalent interactions within starch granules, thereby modulating solubility, pasting behavior, rheological characteristics, and additional functional attributes of starch. This method maintains the ambient temperature, thereby reducing the flavor changes caused by the Maillard reaction and maximizing the retention of nutrients [14]. Existing studies indicate that, compared with conventional thermal treatment or osmotic dehydration (OD) alone, HHP-assisted OD not only achieves rapid dehydration and extends shelf life, but also increases the contents of antioxidants and aromatic compounds in foods. However, when the treatment pressure exceeds 400 MPa, starch granules undergo gelatinization, which in turn inhibits the mass transfer process [15]. Liu et al. [16] showed that low pressure destroyed the structure of starch and protein and increased the interaction between molecules, while high pressure led to protein aggregation and starch gelatinization.

In recent years, studies have begun to focus on the effects of multiple components on starch. For example, Kang et al. [17] reported that the addition of protein and fatty acids promoted the formation of starch–lipid complexes, with different fatty acids exerting varying effects. Zeng et al. [18] demonstrated that β-glucan and protein added to whole-wheat bread exerted a synergistic effect, which stabilized the gluten network and reduced starch digestibility. However, research on the synergistic modification of HHP treatment and ternary complexes remains very limited. In addition, previous studies have shown that at lower pressure levels (100–300 MPa), the pressure has a compression toughening effect on oat starch, enhancing intermolecular interactions. In contrast, when pressure is increased to a higher range (400–600 MPa), it generally leads to the disruption of starch crystalline structure, polymorphic transformation (from A- to V-type), and thorough gelatinization [19]. Therefore, it is hypothesized that the HHP treatment of 300 MPa will promote the formation of the ternary composite system of oat starch/protein/β-glucan, thereby affecting the intercomponent interactions and starch properties.

Therefore, in this study, 300 MPa was chosen as the pressure condition for preparing the ternary composite system. Starch, β-glucan, and protein were isolated from naked oats. Then, according to the content ranges of β-glucan (3–9%) and protein (11–16%) in natural oats, 10% oat β-glucan and 0–30% oat protein were added based on the dry weight of oat starch (100%). This study systematically investigated the effects of the ternary composite system and its interactions on the gel properties, pasting properties, multi-scale structure, and thermal properties of OS under HHP treatment at 300 MPa, providing new insights into the HHP preparation of multi-component oat composite systems and their application in oat starch-based functional foods.

## 2. Results and Discussion

### 2.1. Effects of HHP Treatment on Solubility and Swelling Power of OSGP Composite Systems

**Figure 1:**
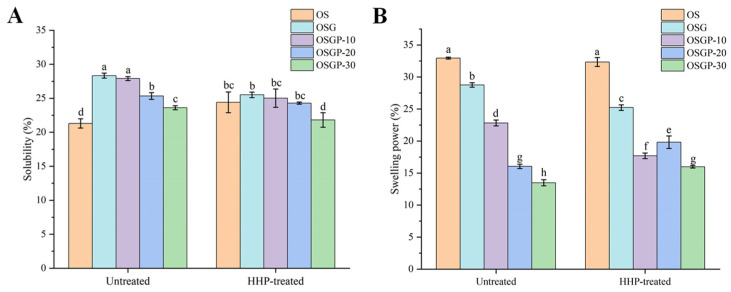
Solubility and swelling power of untreated and HHP-treated OSGP composite systems. (**A**), solubility; (**B**), swelling power. Different superscript letters indicate significant differences (*p* < 0.05).

Starch solubility and swelling power indicate the extent of the interactions between starch and water molecules. As shown in Figure 1A, the solubility of all the composite systems was significantly higher than that of pure starch (*p* < 0.05), and gradually decreased with increasing OP addition. After HHP treatment, the solubility of the composite systems was lower than that of the untreated groups. Compared with pure starch, the increased solubility of the composite systems may be attributable to the introduction of soluble substances like OBG. However, protein competes with starch for water molecules, and the coating effect of high concentrations of protein on starch ultimately diminishes the solubility of the composite system as protein concentration increases [20]. The lower solubility of the HHP-treated composite systems compared to the untreated group suggests that HHP treatment strengthened the ternary interactions and facilitated complex formation, thus decreasing free soluble components and suppressing both water absorption and amylose leaching [21].

As shown in Figure 1B, the swelling power of the composite systems decreased significantly with the increase in OP addition (*p* < 0.05). This may be attributed to the water competition among OBG, OP, and OS, which reduces the available free water content for starch and consequently inhibits its full swelling [22]. Furthermore, Shao et al. [23] reported that proteins can also restrict the swelling of starch granules through electrostatic interactions. The swelling power of the composite systems decreased after HHP treatment, indicating that the 300 MPa HHP treatment enhanced the interactions among the ternary components, promoted the formation of more compact complexes, and inhibited starch swelling.

### 2.2. Effects of HHP Treatment on Gel Properties of OSGP Composite Systems

#### 2.2.1. Freeze–Thaw Stability

**Figure 2:**
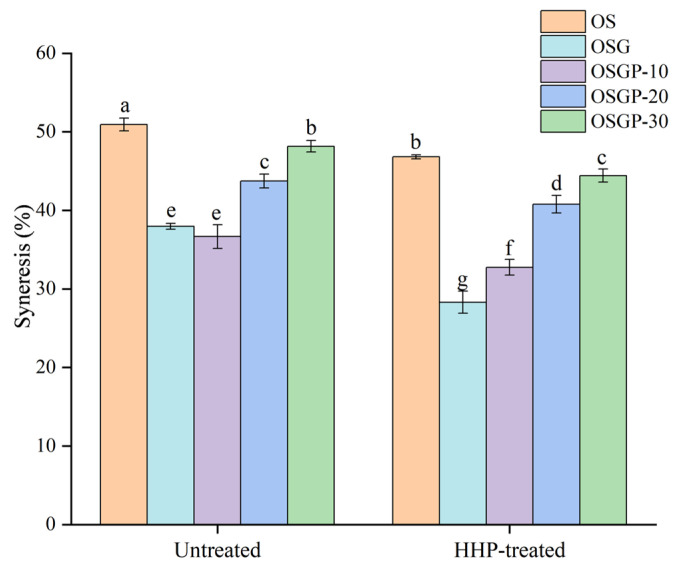
Freeze–thaw stability of untreated and HHP-treated OSGP composite systems. Different superscript letters indicate significant differences (*p* < 0.05).

Freeze–thaw stability was characterized by the syneresis rate, which exhibits an inverse correlation with stability. As shown in Figure 2, syneresis values for HHP-treated samples were lower than those for the untreated control. This may be attributed to the compressional toughening effect of 300 MPa high pressure on starch molecules, which strengthens intermolecular interactions [8], thereby enhancing water-holding capacity and reducing syneresis. The addition of OBG and OP significantly reduced the water evolution rates (*p* < 0.05), because OBG has a high water-holding capacity, and it will reduce the mobility of the starch chain after absorbing water, which will lead to a decrease in recrystallization degree and syneresis degree [24]. In addition, the interaction of OBG, OP, and OS would hinder the recrystallization of starch, reduce the precipitation of water, and improve the freeze–thaw stability. HHP treatment would enhance the interaction of OBG, OP, and OS, and synergistically reduce the water-evolution rate. Wang et al. [25] reported that catechin-chestnut starch complexes exhibited lower syneresis than native chestnut starch when treated at 600 MPa, which was attributed to catechin-induced inhibition of molecular rearrangement and retardation of retrogradation, thereby enhancing water retention and freeze–thaw stability. Fu et al. [26] reported that mulberry leaf polysaccharide reduced syneresis in the mulberry leaf polysaccharide–wheat starch complex, thereby improving freeze–thaw stability. Therefore, HHP treatment, the introduction of polysaccharides and proteins can significantly improve the stability of starch gel by inhibiting recrystallization and water migration. These treatments are conducive to the stability of frozen food and improve product quality.

#### 2.2.2. Texture Profile Analysis (TPA) of the Gel

Table 1 shows that the incorporation of OBG and OP significantly reduced the hardness, gumminess, and chewiness of starch gels (*p* < 0.05), with these properties decreasing as OP concentration increased. The hardness of a starch gel is related to the content of amylose. The higher the content of amylose, the higher the degree of crosslinking and winding between molecules, and the greater the hardness of starch gel [27]. The addition of OBG and OP hindered the rearrangement of amylose molecules and weakened their interactions. Furthermore, the incorporation of protein reduced the effective amylose concentration in the mixture, weakened intermolecular interactions, and thereby hindered the formation of a three-dimensional gel network, resulting in decreased gel hardness, gumminess, and chewiness, as well as delayed retrogradation. In pea starch systems, peanut protein isolate reduced gel hardness [28], while polysaccharide-induced hardness reduction was also observed in wheat starch gels containing mulberry leaf polysaccharides [26]. In contrast, HHP treatment disrupted starch structure and reduced hardness and other texture properties of the starch gel. HHP could cause the unfolding of proteins by denaturation, which adhered to starch granules, and inhibited their swelling, reducing the hardness of the starch gel to a certain extent.

As presented in Table 1, the incorporation of OBG and OP significantly enhanced the springiness and resilience of starch gels (*p* < 0.05). The three-dimensional network structure formed by an appropriate amount of OS and OBG through hydrogen bonding enhances the springiness and resilience of starch gel [29]. While excessive protein would hinder the hydrogen bonding between polysaccharide and starch, resulting in a slight decrease in springiness, the addition of protein would. The HHP treatment caused protein denaturation and unfolding, forming a dense barrier and exacerbating the declining trend in springiness. The experimental results align with those reported by Zhang et al. [30] who demonstrated that the addition of Huangjing polysaccharide (HJP) modulated the interaction between HJP molecules and starch granules, resulting in reduced hardness and chewiness, alongside enhanced springiness. These results indicated that HHP treatment, OBG, and OP significantly affected the texture properties of oat starch gels, inhibited aging, and improved the quality of starch-based foods.

### 2.3. Effects of HHP Treatment on Pasting Properties of OSGP Composite Systems

**Figure 3:**
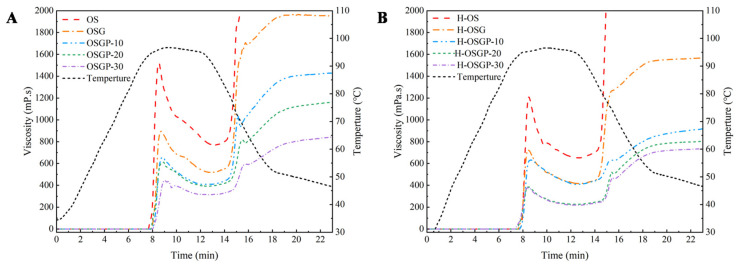
RVA pasting profiles of untreated and HHP-treated OSGP composite systems. (**A**), untreated; (**B**) HHP-treated.

The pasting properties reflect the interactions between starch molecules and water during the pasting process. During the pasting process, water molecules infiltrated into starch microcrystals and destroyed intramolecular hydrogen bonds within the starch molecules [31]. The pasting profiles of samples before and after HHP treatment are presented in Figure 3A and Figure 3B, respectively, with corresponding pasting parameters for all samples summarized in Table 2. From Figure 3, it can be seen that the gelatinization curves of the mixtures moved downward as a whole, and the higher the protein concentration, the greater the downward shift in the curve. Table 2 demonstrates that the addition of OBG and OP reduced the PV, TV, FV, BD, and SB of starch (*p* < 0.05). The presence of OBG and OP competes with starch for water molecules, inducing a transition in the dispersion state of starch granules from an ordered to a disordered arrangement in aqueous medium, thereby inhibiting starch swelling and reducing viscosity. OBG’s high water retention capacity decreases free water content in the system, weakens starch molecular rearrangement and aggregation, and consequently reduces both viscosity and retrogradation degree, consistent with prior research [8]. Proteins bind to or distribute between starch granules through hydrogen bonds, electrostatic forces, and van der Waals forces [32], inhibiting the swelling of starch molecules and the dissolution of amylose, resulting in an incomplete gelatinization process, so that the viscosity decreases with the increase in protein addition.

The BD value reflects the difficulty of the disintegration of soluble starch granules. The BD value decreased significantly after adding OBG and OP, indicating that OBG and OP enhanced the stability of starch molecules against shear damage [33]. The SB value, indicative of short-term retrogradation in starch paste, decreased significantly following the addition of OBG and OP, indicating that these components hindered amylose rearrangement and reduced retrogradation [34]. OBG is bound to the dissolved amylose by hydrogen bonding, which hinders the hydrogen bonding between starch molecules and weakens the aggregation of starch molecules, resulting in a decrease in SB [35]. The addition of OP would reduce the effective starch concentration in the system, thereby decreasing the probability of molecular collisions and inhibiting short-term starch retrogradation. A similar finding was observed in the study of high-amylose corn starch/wheat flour, where the addition of high-amylose corn starch reduced the relative proportion of amylopectin through a dilution effect, thereby decreasing the FV, BD, and SB values [36].

HHP treatment reduced the pasting index of starch and complexes, primarily due to the disruption of starch crystalline structures by high pressure, resulting in a transformation of the disordered state of starch molecules and weakening their interactions. The toughening effect of pressure caused the swelling of starch granules to be limited during gelatinization, thereby reducing the viscosity, which is consistent with the previous research [8]. The HHP treatment also caused protein denaturation and unfolding, as the protein could form a barrier on the starch surface through electrostatic force, which hinders the dissolution of amylose and the recombination of starch molecules, resulting in lower viscosity.

### 2.4. Effects of HHP Treatment on Structural Properties of OSGP Composite Systems

#### 2.4.1. SEM

**Figure 4:**
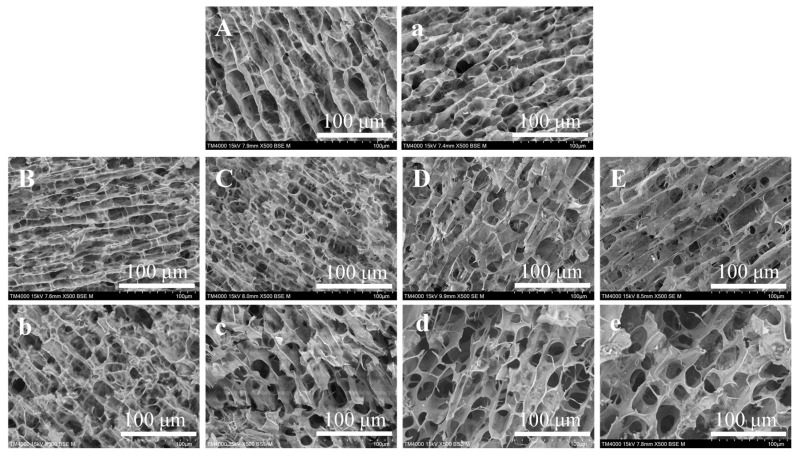
SEM micrographs of cross-sections of OS gels and OSGP composite systems. (magnification: ×500; scale bar: 100 μm). (**A**–**E**) Untreated systems: (**A**) Pure oat starch gel (OS), (**B**) OS-β-glucan complex gel (OSG), (**C**–**E**) OS-β-glucan-protein complex gels with 10–30% protein (OSGP-10/20/30). (**a**–**e**) HHP-treated: (**a**) H-OS, (**b**) H-OSG, (**c**) H-OSGP-10, (**d**) H-OSGP-20, (**e**) H-OSGP-30.

Figure 4 shows the SEM images of all sample gels. All samples exhibited a honeycomb-like three-dimensional network structure, with significant variations in pore size and distribution uniformity observed. These structural differences were attributed to starch molecular rearrangement and water dynamics during freeze-drying [37]. The OS gel has a uniform porous network structure (Figure 4A). Incorporation of OBG reduced gel porosity, thereby forming a uniform, continuous, and dense network structure (Figure 4B). The pores in the gels were produced by the evaporation of water during freeze-drying [31]. The reduction in porosity is attributable to competitive water binding between OBG and starch, impeding water penetration into the starch granule interior and reducing the water available for ice crystal formation [8]. Furthermore, studies have shown that OBG could fill in the gaps between starch granules and interact with starch molecules through hydrogen bonding to form a stable network structure [8]. Similarly, Zhang et al. [30] reported that Huangjing polysaccharide could coat starch granules, inhibit swelling, and act as intergranular bridges to facilitate the generation of more stable gel networks. OBG wrapped around starch granules, inhibits starch swelling during pasting, and effectively reduces the aggregation and arrangement of molecules in the gel structure formed after freeze-drying, thereby forming a dense and uniform gel network [38].

However, the addition of OP significantly altered the microstructure of starch gels (Figure 4C–E). Compared with pure starch gel, the gel containing OBG and OP showed increased pore size, decreased number of pores, and irregular shape. As the OP addition increased, the continuity and uniformity of pore distribution decreased, leading to a less cohesive three-dimensional network structure. This indicates that OP limits the cross-linking between starch molecular chains [39]. OP addition not only inhibited amylose leaching but also hindered the formation of the three-dimensional gel network. Concurrently, steric hindrance arising from OP binding to starch granule surfaces further impeded network formation. This is similar to the study of Niu et al. [40]. HHP treatment strengthened this inhibition. After HHP treatment, the network structure of the gel was more irregular, and the pores were enlarged (Figure 4a–e). This was because HHP treatment destroys the crystal structure of starch and induces protein denaturation and aggregation. The protein aggregates would hinder the uniform growth of ice crystals during the freeze-drying process, and the space they occupy would also form large pores after the ice crystals sublimate. Furthermore, HHP treatment promoted interactions among OBG, OP, and starch molecules, consequently suppressing cross-linking between starch chains and significantly hindering the formation of a compact and homogeneous three-dimensional gel network.

#### 2.4.2. Short-Range Ordered Structure by FT-IR

**Figure 5:**
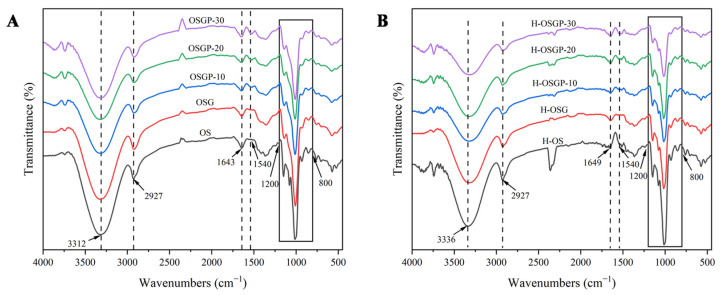
FT-IR spectra of gelatinized OS and OSGP composite systems. (**A**), untreated; (**B**), HHP-treated.

The FTIR spectra (4000–400 cm^−1^) for the samples before and after HHP treatment are presented in Figure 5A and Figure 5B, respectively, while Table 3 summarizes the R_1047/1022_ and R_995/1022_ ratios for all samples. The results in Figure 5 showed that no new absorption peak appeared in any sample, and no characteristic peak disappeared, indicating that no chemical bond modification or covalent bond formation occurred [41], which also indicated that HHP treatment induced physical rather than chemical modification. The bands at 3312 cm^−1^ and 2927 cm^−1^ are assigned to –OH stretching vibration and –CH2 asymmetric stretching vibration, respectively. The incorporation of OBG caused a broadening of the peak at 3312 cm^−1^, indicating that it has a multi-hydroxyl structure. The absorption peak at 1643 cm^−1^ is related to the O–H stretching vibration of bound water in the amorphous region of starch [42]. The absorption peak at about 1540 cm^−1^ was observed in the spectrum after adding protein, which is a typical protein band, belonging to the amide II band (1480–1580 cm^−1^) [43].

In the infrared spectrum, 800–1200 cm^−1^ constitutes the fingerprint region for starch, and the absorption bands at 1047 cm^−1^ and 1022 cm^−1^ correspond to ordered and amorphous starch structures, respectively. R_1047/1022_ and R_995/1022_ are usually used to reflect the degree of order (DO) and the degree of double helix (DD) of starch [44]. Table 3 demonstrates that the incorporation of OBG and OP elevated the R_1047/1022_ value, indicating that OBG and an appropriate amount of OP enhanced the development of short-range ordered starch domains to some extent. Xiao et al. [45] demonstrated that mesona chinensis polysaccharides promote molecular cross-linking and rearrangement in waxy maize starch, thereby enhancing the network structure—similarly, Shao et al. [23] reported that yam protein partially promoted the development of short-range ordered structure in starch. OBG is a linear polysaccharide that can bind to starch through hydrogen bonding to protect the crystalline region of OS [8], while the amino acid residues of OP can form hydrogen bonds or hydrophobic interactions with the hydroxyl groups of OS and OBG, which increases the short-range order of starch. HHP treatment increased the R_1047/1022_ values of the samples, which may be because the HHP treatment enhanced the interaction between OBG, OP, and OS, and the pressure may cause the sample particles to form a more compact composite structure. As shown in Table 3, the addition of OBG, OP, and HHP treatment reduced the R_995/1022_ value, indicating that the double helix structure formed by the leached amylose was reduced or the original double helix structure was destroyed. This phenomenon is attributed to competitive water binding among polysaccharides, proteins, and starch, which weakens the hydrogen bonding network between starch molecules, thereby disrupting starch crystallization regions during gelatinization [30]. Interactions between OBG, OP, and OS, along with the coating effect on starch molecules [30], impede the formation or maintenance of double-helical structures. HHP treatment induces partial unwinding of helices through mechanical disruption of intrachain and interchain hydrogen bonds in starch. Concurrently, HHP treatment will enhance the interaction between OBG, OP, and starch, further preventing the re-formation of the double helix, resulting in a decrease in the content of the double helix structure after HHP treatment.

#### 2.4.3. X-Ray Diffraction (XRD)

**Figure 6:**
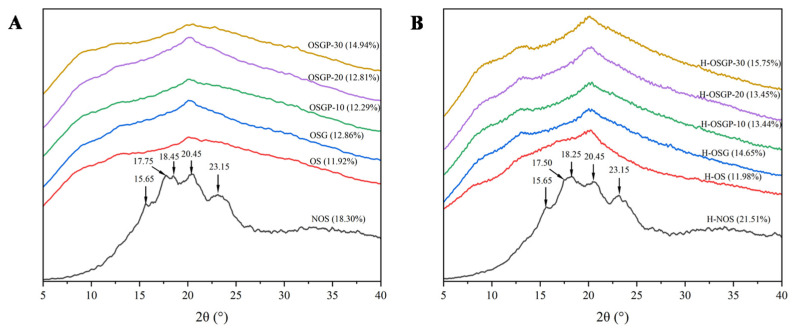
X-ray diffraction patterns of natural OS, gelatinized OS, and OSGP composite systems. (**A**), untreated; (**B**), HHP-treated. Numbers in parentheses are the relative crystallinity based on the peak area.

Figure 6A,B depicts the X-ray diffraction profiles and corresponding relative crystallinity values of natural OS as well as gelatinized samples before and after HHP treatment. As depicted in Figure 6, natural OS exhibited a characteristic A-type crystallographic structure, featuring distinct diffraction peaks located at 15.65°, 17.75°, 18.45°, 20.45°, and 23.15° (2θ), corresponding to a relative crystallinity of 18.30%. The HHP-treated natural OS also showed an A-type structure, and the characteristic peaks did not change significantly, but the relative crystallinity increased to 21.51%. Combined with infrared spectroscopic analysis, these results indicated that HHP treatment enhanced the interaction between starch molecules and increased short-range order [19]. Following gelatinization, the crystal structure was disrupted, evidenced by the disappearance of the A-type characteristic diffraction peak and the emergence of a diffraction peak at approximately 20°, indicating a transition from A-type to V-type crystallinity. In addition, the diffraction peaks became blunted and widened, attributed to the disruption of the crystalline region and partially ordered structure of starch during gelatinization, resulting in significantly lower relative crystallinity for the gelatinized sample compared to natural OS [46]. The addition of OBG and OP increased the relative crystallinity of starch, indicating an interaction among β-glucan, protein, and starch molecules [47]. This may arise from hydrogen bonding between OBG and starch molecules, which confers a protective effect on starch crystallinity [8]. Protein binding with β-glucan and starch suppresses granule swelling during gelatinization. The relative crystallinity increases after HHP treatment, which may be because HHP destroyed the amorphous region, while the interaction between OBG, OP, and OS protected part of the crystalline region, so that the relative proportion of the crystalline part increased, that is, the relative crystallinity increases. This result was consistent with previous studies [48], which could be attributed to the disruption of hydrogen bonds and starch chain architectures induced by HHP treatment, thereby causing the disintegration of amorphous domains, while the interaction among polysaccharides, proteins, and starch protects some crystalline regions. HHP treatment also enhanced the interaction between the three and, therefore, promoted the increase in relative crystallinity and the formation of short-range ordered structure.

### 2.5. Effects of HHP Treatment on Thermal Properties of OSGP Composite Systems

The thermodynamic parameters of the samples before and after HHP treatment are shown in Table 4. Compared with the starch system alone, To, Tp, and Tc of the composite systems with OBG and OP increased, but the ΔH decreased significantly (*p* < 0.05). This indicated that the incorporation of OBG and OP modulated the thermal properties of starch. Zhuang et al. [49] reported that Lentinus edodes β-glucan could increase the gelatinization temperature of wheat starch. This may be because the high hydrophilicity of polysaccharides and proteins could reduce the hydration of starch and delay starch gelatinization. Oppong et al. [50] suggested that proteins inhibited starch gelatinization mainly by increasing To and Tp of starch. Luo et al. [51] demonstrated that the weakening of water-starch hydrogen bonding could reduce the breakage of hydrogen bonds between starch molecules, suppress starch granule swelling, and result in an elevation of the starch gelatinization temperature.

The ΔH of starch characterizes the energy required to destroy its double helix structure. Table 4 shows reduced starch ΔH values with the incorporation of OBG and OP, thus suggesting that both suppressed amylose rearrangement, which was consistent with the decrease in the SB value of RVA. Polysaccharides can reduce ΔH by limiting the migration of water to the amorphous region of starch granules [30]. Similarly, Yang et al. [52] suggested that proteins may reduce ΔH by interfering with water migration into starch granules during gelatinization and weakening starch–water interactions. In addition, the addition of OBG and OP reduced the relative concentration of starch in the mixture, and its dilution effect also reduced ΔH. The further decrease in ΔH after HHP treatment indicates that HHP treatment promoted the interactions among the ternary components, enhanced the formation of the composite system, and further restricted water migration into the starch granules. Zhou et al. [53] found that HHP treatment can partially unfold proteins and enhance the interaction between proteins and water molecules. Liu et al. [16] showed that HHP treatment strengthens the interaction between starch and protein, which is the reason for the decrease in ΔH of starch-protein complexes. Furthermore, HHP-induced protein denaturation and unfolding can further enhance its steric hindrance effect and delayed starch gelatinization. In conclusion, HHP treatment and the addition of OBG and OP can reduce the ΔH value of oat starch and delay the gelatinization process.

## 3. Conclusions

Starch, β-glucan, and protein constitute the primary components of oats, with their interactions critically influencing the physicochemical properties of oat-based systems. This study systematically investigated the interactions among oat starch (OS), β-glucan (OBG), and protein (OP) under high hydrostatic pressure (HHP) treatment and their effects on the pasting properties, gel characteristics, structural features, and thermal behavior of starch. Under ambient pressure, OBG and OP primarily restricted starch granule swelling through water competition, resulting in decreased swelling power, paste viscosity, relative crystallinity, and gelatinization enthalpy (ΔH). They bind to starch through hydrogen bonding and other non-covalent interactions, thereby inhibiting the reassociation of starch molecules, delaying the gelatinization process, and impeding the short-term retrogradation of starch. Texture profile analysis revealed that the incorporation of OBG and OP diminished the hardness, gumminess, and chewiness while enhancing springiness and resilience in starch gels. After 300 MPa HHP treatment, the interactions among the three components were significantly enhanced, with hydrogen bonding being the predominant force. HHP disrupted starch amorphous regions, increased crystalline domains proportion, and consequently elevated the system’s relative crystallinity and short-range order of the system, although the double-helical content decreased. Furthermore, HHP improved the freeze–thaw stability, gel elasticity, and thermal stability of the composite system, but was accompanied by reductions in solubility, paste viscosity, and gelatinization enthalpy. This study clarifies the mechanisms by which OBG and OP influence starch properties under ambient conditions through water competition and intermolecular binding, and reveals how HHP modulates the functional properties of starch by intensifying ternary interactions. The findings establish a theoretical foundation for elucidating structure-function relationships in multicomponent oat systems under non-thermal processing, thereby facilitating the development of novel healthy functional foods. However, this study investigated the effect of HHP on the composite system using a reconstitution approach, which neglected the in situ binding interactions of non-starch components within the native oat kernel. Therefore, in future studies, selective removal of endogenous β-glucan or protein from the native kernel could be employed to further investigate the mechanism of action among multiple non-starch components under HHP and their impact on starch properties.

## 4. Materials and Methods

### 4.1. Materials

Naked oats were procured from Inner Mongolia Xibei Huitong Agricultural Technology Development Co., Ltd. (Hohhot, China). Neutral protease (50 U/mg) was purchased from Shanghai Macklin Biochemical Technology Co., Ltd. (Shanghai, China). Thermostable α-amylase (50 U/mg) was obtained from Beijing Solarbio Science & Technology Co., Ltd. (Beijing, China). Megazyme (Megazyme International Ireland, Bray, Co., Wicklow, Ireland) supplied the β-Glucan Assay Kit (Mixed Linkage). The remaining chemical reagents used were analytical grade.

### 4.2. Preparation of Samples

#### 4.2.1. Extraction of Oat Starch

OS was extracted using the method of Zhang et al. [8] with minor modifications. Defatted oat flour was prepared by removing the fat from oat flour (80 mesh) with petroleum ether. The defatted oat flour was added to the NaOH solution at a ratio of 1:10 (*w*/*v*), and the pH was adjusted to 10.0, stirring at 35 °C for 2 h. The suspension was centrifuged at 4000× *g* for 15 min, and the supernatant, together with the gray upper tissue, was removed. The white precipitate was washed repeatedly using distilled water until there was no gray precipitate in the upper layer. After the white precipitate was dried, the protein was removed with a neutral protease. The purified oat starch was freeze-dried and crushed (starch 93.05%, protein 0.32%, fat 1.24%).

#### 4.2.2. Extraction of the Oat β-Glucan

OBG was extracted, referring to the method reported by Kim et al. [54] with some modifications. Oat bran powder (60 mesh) was refluxed with 80% ethanol (1:10, *w*/*v*) at 90 °C for 2 h to eliminate fat-soluble compounds and inactivate endogenous enzymes. The dried oat bran powder was subsequently suspended in distilled water (1:20, *w*/*v*) and agitated at 80 °C for 2 h. The suspension was centrifuged at 4500× *g* for 15 min, and the supernatant was collected. To this supernatant, 0.5% thermostable α-amylase was added, followed by incubation in a water bath at 90 °C for 1 h until no blue color developed upon iodine solution addition. The pH of the extract was adjusted to 4.5 using 2 mol/L HCl, and the mixture was stored at 4 °C overnight. After re-centrifuging, the supernatant was adjusted to pH 7.0, concentrated to one-third of its initial volume, supplemented with three volumes of absolute ethanol, and stored at 4 °C overnight. The precipitate was collected by centrifugation, redissolved in water, and deproteinized by enzymatic treatment combined with the Sevag method. The deproteinized solution was then supplemented with three volumes of absolute ethanol and stored at 4 °C overnight. The resulting precipitate was isolated by centrifugation and washed sequentially with acetone and anhydrous ethanol, dissolved in water, dialyzed for 3 days, and then freeze-dried. The content of β-glucan was 80.72% (*w*/*w*), measured by the β-Glucan Assay Kit (Mixed Linkage).

#### 4.2.3. Extraction of Oat Protein

OP was extracted, referring to the method reported by Qian et al. [39] with minor modifications. Defatted oat flour was suspended in distilled water (1:20, *w*/*v*), and the pH was adjusted to 10.0 using NaOH, followed by agitation at 45 °C for 2 h. The resulting suspension was subjected to centrifugation at 4000× *g* for 15 min, and the supernatant was retained. Following adjustment of the supernatant pH to 4.5 with HCl, the solution was maintained at 4 °C for 30 min and subjected to centrifugation. The precipitate was washed three times with distilled water and centrifuged after each wash. The final precipitate was reconstituted in water, adjusted to pH 7.0, and lyophilized. Protein content, quantified by the Kjeldahl method, was 92.45% (*w*/*w*).

#### 4.2.4. Preparation and High Hydrostatic Pressure Treatment of Oat β-Glucan-Protein-Starch Complex Systems

Based on the OS dry weight (100%), OBG was added at 10% (*w*/*w*) of the starch dry weight, and OP was added at 0%, 10%, 20%, and 30% (*w*/*w*) of the starch dry weight, respectively. These ratios were based on the content ranges of β-glucan and protein in natural oats, aiming to simulate the proportional relationships among real oat components.

OBG was added to distilled water and stirred at 80 °C for 15 min to ensure complete dissolution. OS was then mixed with OP in the aforementioned ratios, and the mixture was slowly added to the cooled OBG solution under continuous stirring for 15 min. Finally, the volume was adjusted with distilled water, yielding a suspension whose total solid concentration was 15% (*w*/*v*).

The prepared suspension was divided into two portions. One portion was vacuum-sealed in polyethylene vacuum bags and treated at 300 MPa for 15 min using high-pressure equipment (HHP-600, Baotou Kefa High Pressure Technology Co., Ltd., Baotou, China). This treatment condition was determined based on previous studies from our laboratory [8,19]. The other portion served as the untreated control group, which was also vacuum-sealed and left to stand at ambient pressure and room temperature for 15 min. All samples (both untreated and treated groups) were immediately freeze-dried after the respective treatments, and the dried samples were crushed and stored in a dryer for analysis.

For simplicity, samples without HHP treatment were designated as follows: oat starch as OS, oat starch with 10% β-glucan and 0%, 10%, 20%, 30% protein (on starch dry basis) as OSG, OSGP-10, OSGP-20, OSGP-30, respectively. The corresponding samples with HHP treatment were designated as H-OS, H-OSG, and H-OSGP10 to 30, respectively.

### 4.3. Solubility and Swelling Power

Solubility and swelling power were determined following the method of Shi et al. [55] with minor modifications. A certain amount of sample (dry basis) was weighed and suspended in distilled water to yield a 2% (*w*/*v*) solution. The suspension was agitated at 95 °C for 30 min in a water bath, cooled to room temperature, and centrifuged at 4000× *g* for 15 min. The supernatant was collected, and the precipitate was weighed. The supernatant was dried to constant weight at 105 °C. Each test was performed three times. The solubility and swelling power were calculated, respectively, using Equations (1) and (2):

S (%) = (A/W) × 100,(1)

SP (%) = (P × 100)/(W(100 − S)),(2)

In the formulas, W, sample mass (g); A, constant weight of supernatant (g); P, weight of sediment (g); S, solubility; SP, swelling power.

### 4.4. Gel Properties

#### 4.4.1. Freeze–Thaw Stability Analysis

The previously reported method was used and slightly modified [14]. An appropriate amount of sample (dry basis) was weighed and suspended in distilled water to yield a 5% (*w*/*v*) suspension. The mixture was subjected to thermostatic oscillation at 95 °C for 30 min, then cooled to ambient temperature before being reweighed and transferred into centrifuge tubes. Following 24 h freezing at −20 °C, samples were thawed (30 °C, 2 h) and centrifuged. The supernatant was decanted, and the precipitate mass was recorded. All measurements for each experimental group were performed in triplicate. The water extraction rate was calculated using Equation (3):

syneresis rate (%) = ((M_1_ − M_2_)/M_1_) × 100, (3)

where M_1_ is the weight of the sample in the centrifuge tube (g), and M_2_ is the weight of the precipitate after centrifugation (g).

#### 4.4.2. Texture Profile Analysis (TPA) of the Gels

A texture analyzer (TA-XT2i, Stable Micro Systems Ltd., Godalming, UK) was used to measure the gel texture, referring to previous methods with minor modifications [56]. An appropriate mass of sample (dry basis) was dispersed in distilled water to prepare a 12% (*w*/*v*) suspension, then stirred in a boiling water bath for 30 min to ensure complete gelatinization. After gelatinization, it was removed, cooled to ambient temperature, and kept at 4 °C for 24 h to reach stability. The P36 probe was used, the compression ratio was 50%, the test speed was 1.0 mm/s, the trigger force was 5.0 g, and the two compression intervals were 3 s. The experiment was repeated three times with freshly prepared samples.

### 4.5. Pasting Properties

Starch pasting properties were evaluated using a rapid viscosity analyzer (FDV-E, Shanghai Nirun Intelligent Technology Co., Ltd., Shanghai, China) following the procedure described by Zhang et al. [8]. An appropriate mass of sample (dry basis) was weighed and dispersed in distilled water to prepare a 7% (*w*/*v*) suspension, which was homogenized to prevent particle agglomeration. The suspension was transferred into an RVA aluminum canister, the temperature program was set, and the pasting curve was recorded to obtain the characteristic parameters.

### 4.6. Structural Properties

#### 4.6.1. Scanning Electron Microscopy (SEM)

The gel samples were obtained from RVA analysis and freeze-dried. The dried samples were examined by scanning electron microscopy (TM 4000, Hitachi High-Tech Corporation, Tokyo, Japan). The acceleration voltage of the SEM is set to 15 kV.

#### 4.6.2. Fourier Transform Infrared Spectroscopy (FTIR)

The method was slightly modified according to Zhang et al. [19]. Freeze-dried gel samples were pulverized, sieved through a 100-mesh screen, blended with potassium bromide at a 1:100 (*w*/*w*) ratio, ground together, and subsequently compressed into thin disks. FT-IR spectra were recorded with a spectrometer (Thermo Fisher Scientific, Waltham, MA, USA) using 64 scans at 4 cm^−1^ resolution over the wavenumber range of 4000–400 cm^−1^.

#### 4.6.3. X-Ray Diffraction (XRD) Analysis

Slight modifications were made to the method according to Zhao et al. [29]. Freeze-dried gel samples were crushed and sieved (100 mesh), followed by crystalline structure analysis using an X-ray diffractometer (Ultima IV, Rigaku Corporation, Tokyo, Japan). XRD measurements were performed with a scanning range of 5–40° (2θ) at a scan rate of 1.5°/min.

### 4.7. Differential Scanning Calorimeter (DSC) Analysis

Thermal properties were analyzed by differential scanning calorimetry (DSC200, Hitachi High-Tech Corporation, Tokyo, Japan) according to Zhang et al. [8] with minor modifications. A 3.0 mg sample was placed in an aluminum crucible, combined with 9 μL deionized water, and hermetically sealed. After overnight equilibration under ambient conditions, the sealed crucible was heated at 10 °C/min from 30 °C to 120 °C. An empty aluminum crucible was used as the reference. The thermal transition parameters were obtained by data acquisition software (NEXTA, v3.2 build 17).

### 4.8. Statistical Analysis

All experiments were repeated at least three times. The experimental data were expressed as mean ± standard deviation. For XRD analysis, a single determination was performed. Origin 2024 was used to generate charts. One-way analysis of variance and sample comparison were conducted using IBM SPSS Statistics 25.0, and significant differences among experimental means were identified using Duncan‘s multiple range test (*p* < 0.05).

## Figures and Tables

**Table 1:** Textural parameters of untreated and HHP-treated OSGP composite systems.

Samples	Hardness (g)	Springiness (mm)	Gumminess (g)	Chewiness (mJ)	Resilience
OS	502.00 ± 2.34 ^a^	3.88 ± 0.09 ^ef^	352.80 ± 3.75 ^a^	19.08 ± 0.21 ^a^	0.26 ± 0.01 ^b^
OSG	241.30 ± 1.94 ^c^	5.24 ± 0.26 ^b^	210.70 ± 5.95 ^c^	10.60 ± 0.52 ^c^	0.44 ± 0.01 ^a^
OSGP-10	195.70 ± 4.38 ^e^	4.24 ± 0.08 ^cd^	183.20 ± 1.70 ^e^	7.61 ± 0.07 ^d^	0.43 ± 0.02 ^a^
OSGP-20	168.50 ± 1.18 ^g^	4.08 ± 0.06 ^de^	154.10 ± 2.39 ^g^	6.16 ± 0.25 ^ef^	0.41 ± 0.02 ^a^
OSGP-30	165.10 ± 1.69 ^h^	4.17 ± 0.19 ^de^	149.20 ± 4.26 ^g^	6.10 ± 0.15 ^f^	0.42 ± 0.01 ^a^
H-OS	401.20 ± 4.14 ^b^	4.54 ± 0.05 ^c^	291.90 ± 1.91 ^b^	12.99 ± 0.03 ^b^	0.30 ± 0.01 ^b^
H-OSG	208.90 ± 2.49 ^d^	5.30 ± 0.38 ^b^	200.20 ± 0.59 ^d^	10.40 ± 0.32 ^c^	0.45 ± 0.01 ^a^
H-OSGP-10	180.70 ± 0.82 ^f^	5.80 ± 0.17 ^a^	162.60 ± 2.61 ^f^	6.62 ± 0.09 ^e^	0.45 ± 0.01 ^a^
H-OSGP-20	161.80 ± 0.90 ^i^	4.07 ± 0.04 ^de^	152.30 ± 1.51 ^g^	5.93 ± 0.23 ^f^	0.48 ± 0.03 ^a^
H-OSGP-30	159.70 ± 1.54 ^j^	3.56 ± 0.10 ^f^	136.30 ± 1.36 ^h^	3.99 ± 0.18 ^g^	0.44 ± 0.00 ^a^

Data are represented as the mean ± SD (*n* = 3). Different superscript letters within columns indicate significant differences (*p* < 0.05).

**Table 2:** Pasting parameters of untreated and HHP-treated OSGP composite systems.

Samples	PV (mPa.s)	TV (mPa.s)	FV (mPa.s)	BD (mPa.s)	SB (mPa.s)
OS	1516.02 ± 7.14 ^a^	768.06 ± 0.26 ^a^	ND	747.96 ± 0.40 ^a^	ND
OSG	896.20 ± 0.73 ^c^	518.10 ± 1.20 ^c^	1953.86 ± 5.27 ^a^	378.11 ± 0.92 ^c^	1435.76 ± 5.27 ^a^
OSGP-10	655.93 ± 1.20 ^e^	409.97 ± 1.12 ^e^	1429.82 ± 0.33 ^c^	245.96 ± 3.09 ^e^	1019.85 ± 1.21 ^c^
OSGP-20	614.00 ± 0.90 ^g^	391.92 ± 3.08 ^g^	1163.72 ± 0.28 ^d^	222.08 ± 0.86 ^g^	771.80 ± 0.20 ^d^
OSGP-30	444.05 ± 1.08 ^h^	313.94 ± 0.81 ^h^	844.03 ± 0.41 ^f^	130.11 ± 0.88 ^i^	530.09 ± 0.40 ^f^
H-OS	1225.60 ± 5.58 ^b^	615.42 ± 0.84 ^b^	ND	610.18 ± 1.27 ^b^	ND
H-OSG	720.81 ± 1.16 ^d^	415.72 ± 1.07 ^d^	1565.40 ± 3.06 ^b^	305.09 ± 1.21 ^d^	1149.68 ± 1.10 ^b^
H-OSGP-10	631.55 ± 1.13 ^f^	408.10 ± 1.05 ^f^	917.91 ± 1.08 ^e^	223.45 ± 1.15 ^f^	509.81 ± 2.06 ^h^
H-OSGP-20	385.14 ± 0.50 ^i^	225.96 ± 0.02 ^i^	803.16 ± 0.45 ^g^	159.18 ± 0.51 ^h^	577.20 ± 0.43 ^e^
H-OSGP-30	377.04 ± 0.42 ^j^	217.70 ± 0.62 ^j^	733.79 ± 0.83 ^h^	159.34 ± 1.02 ^h^	516.09 ± 0.21 ^g^

PV, peak viscosity; TV, trough viscosity; FV, final viscosity; BD, breakdown viscosity; SB, setback viscosity. ND, not detected. Data are represented as the mean ± SD (*n* = 3). Different superscript letters within columns indicate significant differences (*p* < 0.05).

**Table 3:** The R_1047/1022_ and R_995/1022_ values of untreated and HHP-treated OSGP composite systems.

Samples	R_1047/1022_	R_995/1022_
OS	0.585 ± 0.011 ^d^	1.025 ± 0.038 ^a^
OSG	0.603 ± 0.006 ^c^	1.013 ± 0.009 ^a^
OSGP-10	0.631 ± 0.007 ^b^	0.931 ± 0.020 ^b^
OSGP-20	0.643 ± 0.016 ^b^	0.933 ± 0.020 ^b^
OSGP-30	0.643 ± 0.008 ^b^	0.928 ± 0.029 ^bc^
H-OS	0.594 ± 0.003 ^cd^	1.020 ± 0.021 ^a^
H-OSG	0.635 ± 0.004 ^b^	0.935 ± 0.014 ^b^
H-OSGP-10	0.660 ± 0.009 ^b^	0.929 ± 0.023 ^b^
H-OSGP-20	0.661 ± 0.007 ^b^	0.869 ± 0.025 ^d^
H-OSGP-30	0.671 ± 0.002 ^a^	0.884 ± 0.007 ^cd^

Data are represented as the mean ± SD (*n* = 3). Different superscript letters within columns indicate significant differences (*p* < 0.05).

**Table 4:** The thermodynamic parameters of untreated and HHP-treated OSGP composite systems.

Samples	T_o_ (°C)	T_p_ (°C)	T_c_ (°C)	ΔH (J/g)
OS	57.07 ± 0.12 ^ef^	63.26 ± 0.47 ^d^	77.93 ± 0.61 ^e^	2.74 ± 0.09 ^a^
OSG	57.21 ± 0.20 ^e^	64.03 ± 0.60 ^c^	79.18 ± 0.84 ^d^	2.42 ± 0.05 ^b^
OSGP-10	57.80 ± 0.08 ^d^	64.91 ± 0.53 ^a^	80.80 ± 1.01 ^c^	2.35 ± 0.04 ^b^
OSGP-20	58.44 ± 0.68 ^bc^	64.79 ± 0.10 ^ab^	81.87 ± 0.11 ^b^	2.18 ± 0.06 ^c^
OSGP-30	58.89 ± 0.22 ^a^	65.04 ± 0.26 ^a^	83.31 ± 0.82 ^a^	1.87 ± 0.21 ^d^
H-OS	56.41 ± 0.42 ^g^	62.55 ± 0.97 ^e^	75.70 ± 0.10 ^g^	2.33 ± 0.03 ^bc^
H-OSG	56.86 ± 0.55 ^f^	62.87 ± 0.23 ^de^	76.45 ± 0.45 ^f^	2.00 ± 0.01 ^d^
H-OSGP-10	57.20 ± 0.42 ^e^	63.14 ± 0.34 ^d^	76.60 ± 0.30 ^f^	1.14 ± 0.04 ^f^
H-OSGP-20	58.17 ± 0.53 ^c^	64.07 ± 0.27 ^c^	77.77 ± 0.22 ^e^	1.60 ± 0.06 ^e^
H-OSGP-30	58.62 ± 0.13 ^ab^	64.35 ± 0.51 ^bc^	79.36 ± 0.32 ^d^	1.51 ± 0.02 ^e^

T_o_, Onset temperature; T_p_, peak pasting temperature; T_c_, conclusion temperature; ΔH, gelatinization enthalpy. Data are represented as the mean ± SD (*n* = 3). Different superscript letters within columns indicate significant differences (*p* < 0.05).

## Data Availability

The original contributions presented in the study are included in the article; further inquiries can be directed to the corresponding author.
